# Low-Dose Ketamine Infusion for Perioperative Pain Management in Patients Undergoing Laparoscopic Gastric Bypass: A Prospective Randomized Controlled Trial

**DOI:** 10.1155/2021/5520517

**Published:** 2021-07-21

**Authors:** Mitchell T. Seman, Shawn H. Malan, Matthew R. Buras, Richard J. Butterfield, Kristi L. Harold, James A. Madura, David M. Rosenfeld, Andrew W. Gorlin

**Affiliations:** ^1^Department of Anesthesiology and Perioperative Medicine, Mayo Clinic Hospital, 5777 E Mayo Blvd, Phoenix, AZ 85054, USA; ^2^Department of Research and Biostatistics, Mayo Clinic Hospital, 5777 E Mayo Blvd, Phoenix, AZ 85054, USA; ^3^Department of General Surgery, Mayo Clinic Hospital, 5777 E Mayo Blvd, Phoenix, AZ 85054, USA; ^4^Department of Anesthesiology and Perioperative Medicine, Mayo Clinic Hospital, 5777 E Mayo Blvd, Phoenix, AZ 85054, USA

## Abstract

**Introduction:**

Obesity is a common comorbidity seen in the perioperative setting and is associated with many diseases including cardiovascular disease and obstructive sleep apnea. Laparoscopic Roux-en-Y gastric bypass is the gold standard surgical treatment for patients whose weight is refractory to diet and exercise. Caring for these patients perioperatively presents unique challenges to anesthesiologists and is associated with an increased risk of adverse respiratory events. In our study, we hypothesize that a low-dose perioperative ketamine infusion will reduce opioid consumption and improve analgesia when compared to standard therapy.

**Methods:**

This is a single-center, prospective randomized controlled study enrolling 35 patients in total. Patients were randomized equally into the ketamine and control group. Preop, intraop, and postop management regimens were standardized. The ketamine group received a 0.3 mg/kg ideal body weight ketamine bolus after induction followed by a 0.2 mg/kg/hr ketamine infusion continued into the postop setting for up to 24 hours. Data collected included total perioperative opioids used converted to oral morphine equivalents (ME), pain scores, side effects, hospital length of stay, and patient satisfaction captured via postoperative questionnaires.

**Results:**

The use of perioperative opioid consumption was significantly lower in the ketamine group when compared with the control group (179.9 ME versus 248.7 ME, *P*=0.03). There was no statistically significant difference in pain scores or hospital length of stay postoperatively between the two groups. There were also no reported adverse respiratory events, prolonged sedation, agitation, or other side effects reported in either group. The patient satisfaction questionnaires showed a significant difference with the ketamine group reporting lower maximum pain scores, a decrease in how pain limited activities of daily living once discharged, and increased hospital pain management satisfaction scores.

**Conclusions:**

Perioperative low-dose ketamine infusions significantly reduced opioid consumption in morbidly obese patients undergoing laparoscopic gastric bypass surgery.

## 1. Introduction

Obesity is a public health crisis in the developed world, especially in the United States. Negative health consequences of obesity include cardiovascular disease, obstructive sleep apnea (OSA), systemic hypertension, pulmonary hypertension, diabetes, musculoskeletal problems, and various malignancies [1]. Given the severe health consequences of obesity and the difficulty that many patients have maintaining a healthy weight, surgical management has become an effective therapeutic option for many patients with morbid obesity [2].

Laparoscopic Roux-en-Y gastric bypass is considered the “gold standard” for weight loss surgery and has a long track record of safety and efficacy. There is an association between obesity and OSA which is a risk factor for perioperative respiratory depression as well as respiratory arrest and death [3]. Up to 70% of patients undergoing bariatric surgery have OSA, and these patients are especially vulnerable to apneic events postoperatively due to surgical stress, residual effects of anesthesia, disruption of the normal sleep cycle, and exposure to central nervous system depressants [4, 5]. Opioids in particular are problematic as they cause respiratory depression and depress the muscle tone of the upper airway [6, 7].

Ketamine is an N-methyl-D-aspartate (NMDA) receptor antagonist that, at high doses (>1 mg/kg), induces dissociative anesthesia with minimal effects on ventilation and airway reflexes [8]. Low-dose ketamine infusions (<0.3 mg/kg/hr) have proven efficacy for reducing pain scores as well as reducing postoperative opioid consumption in a wide variety of surgical procedures [8, 9]. Furthermore, even at anesthetic doses, ketamine has no negative effect on pharyngeal muscle tone [10]. For these reasons, low-dose ketamine may be an ideal analgesic adjunct for patients undergoing gastric bypass.

In this randomized, prospective trial, we compare low-dose intra- and postoperative ketamine infusion with standard analgesia in patients undergoing laparoscopic gastric bypass. Our hypothesis is that perioperative ketamine infusion will reduce opioid consumption and improve analgesia. The primary outcome is opioid consumption (in oral morphine equivalents). Secondary outcomes include pain scores, ketamine-related adverse events, respiratory depression, sedation, length of stay, and patient satisfaction.

## 2. Methods

### 2.1. Study Population

This prospective, randomized study was approved by our Institutional Review Board (IRB 17–000301). Funding for this study came from a small intramural grant, and all methods were performed in accordance with the relevant guidelines and regulations. The study was not blinded, and neither were the outcome assessors as funding for this could not be obtained. The study was designed to enroll thirty-four patients, seventeen to each therapy group.

Written informed consent was obtained from all study participants. The study was performed from August 2018 to December 2019. Surgeon appointment calendars were screened for patients that met criteria for the study. Those eligible were consented by a physician or study coordinator in either the preoperative evaluation clinic or the surgery clinic. Inclusion criteria included age ≥18 years and <70 years old undergoing laparoscopic gastric bypass surgery at the BMI ≥35 consent obtained as per policy. Exclusion criteria included intolerance or allergy to ketamine; history of schizophrenia, schizoaffective disorder or other psychiatric diagnoses with psychotic features; presence of unstable cardiovascular disease including the presence of acute coronary syndrome, unstable angina, hypertensive emergency, acute TIA, or stroke; presence of acute elevation of intracranial or intraocular pressure; presence of seizure disorder, history of substance abuse or addiction, and creatinine >1.5; end-stage liver disease; and pregnancy. Patients with chronic pain and/or chronic opioid therapy were not excluded from this study in order to more closely replicate the study population of interest; however, we did exclude patients taking >50 oral morphine equivalents per day for more than one month prior to surgery.

### 2.2. Study Protocol

Patients were randomized to either the standard therapy group or the ketamine therapy group after consent was obtained using the REDcap (Research Electronic Data Capture tool). For each group, the patient's ideal body weight (IBW) was calculated using an online calculator (http://globalrph.com/ibw_calc.htm). Patients randomized to the standard therapy and ketamine therapy groups study protocol received preoperative dexamethasone 4 mg IV and midazolam 1–2 mg intravenous (IV) for anxiolysis at the discretion of the supervising anesthesiologist. Supervising anesthesiologists, nurse anesthetists, and residents varied greatly as it was difficult logistically to have the same providers caring for each study patient.

Induction of anesthesia was achieved using propofol 2–3 mg/kg IV (IBW) and fentanyl 1 mcg/kg IV (IBW). Neuromuscular blockade was achieved with succinylcholine and/or rocuronium and maintained with rocuronium. All patients had an endotracheal tube placed. Sevoflurane was used for maintenance of anesthesia. Additional doses of fentanyl 0.5–1 mcg/kg (IBW) were used for intraoperative analgesia at the discretion of the supervising anesthesiologist. Upon completion of the surgery, the patient received acetaminophen 1 g IV, ketorolac 30 mg IV, and ondansetron 4 mg IV unless contraindicated. Reversal of neuromuscular blockade was achieved with Sugammadex 2–4 mg/kg (actual body weight) depending on twitch response per drug manufacturer's recommended protocol. Postoperative analgesia included a hydromorphone patient controlled analgesia pump (PCA) programmed to deliver 0.2 mg IV every 8 minutes if requested by the patient, acetaminophen 1 g IV every 8 hours for 3 doses, and ketorolac 15 mg IV every 6 hours for 3 doses. Other postoperative care was managed by the surgical team as per routine.

Patients randomized to the ketamine therapy group received a ketamine bolus (0.3 mg/kg IV IBW) with induction; a ketamine infusion (0.2 mg/kg/hr IBW) was started after induction and terminated the next morning after surgical team rounds or at 24 hours. The ketamine infusion was not titrated but could be terminated if the patient was having side-effects relating to the drug; the pain service followed all patients receiving a ketamine infusion to assist with troubleshooting and determining the need for infusion termination. The remainder of the care was identical to the standard therapy group.

### 2.3. Data Collection

The primary study outcome is to determine the total cumulative perioperative opioid dose measured in oral morphine equivalents from induction of anesthesia until 48 hours after induction of anesthesia (calculated using the advanced GlobalRPH tool https://globalrph.com/medcalcs/opioid-pain-management-converter-advanced/). Secondary study outcomes included visual analog pain scores measured from PACU arrival until 48 hours after induction of anesthesia. Postoperative nausea/vomiting and pruritis were recorded from PACU arrival until discharge from hospital; hospital length of stay and patient satisfaction with pain management both at time of discharge and at the postoperative visit were measured by questionnaires completed by the patient at these time points. Primary safety outcomes included careful and continuous monitoring, intervention and recording of hemodynamics, sedation, agitation, hallucinations, delirium, and adverse respiratory events (unexpected initiation of noninvasive ventilation, naloxone administration, or SPO2 <90%). Standardized nursing procedures were used including vital signs and sedation scores, according to standard intensive postsurgical requirements.

### 2.4. Statistical Analysis

A sample size of seventeen in each group will have 80% power to detect a probability of 0.746 that an observation in the new management group is less than an observation in the control group using a Wilcoxon (Mann–Whitney) rank-sum test with a 0.050 one-sided significance level. Continuous variables were compared between ketamine and control groups using the equal variance *t*-test or Wilcoxon rank-sum test, where appropriate, while categorical variables were compared using the chi-square test. All hypothesis tests were two-sided with *P* < 0.05 considered statistically significant. All analyses were conducted in SAS v9.4 (SAS Institute, Inc.; Cary, NC).

## 3. Results

**Figure 1:**
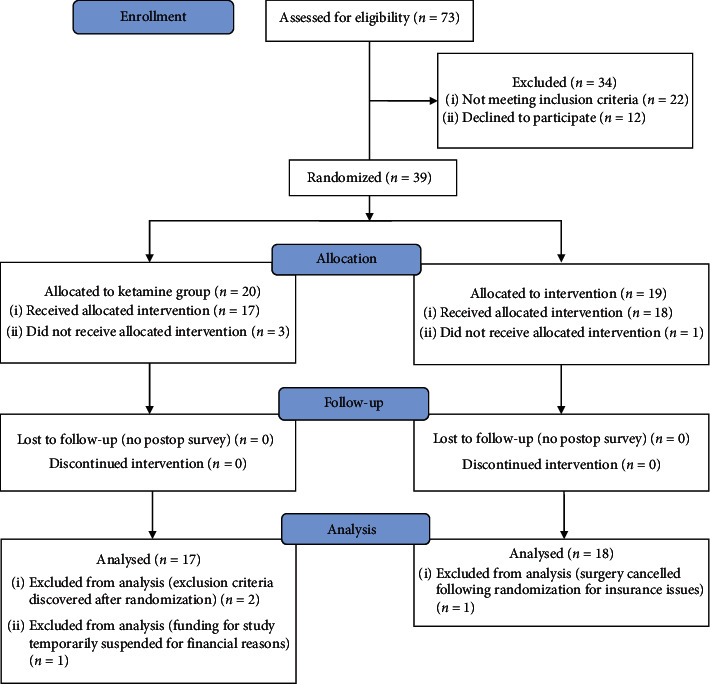
CONSORT flow diagram illustrating patient enrollment through analysis of the data.

A total of thirty-nine patients undergoing elective laparoscopic gastric bypass surgery were enrolled into the study. Nineteen patients were randomized into the standard of care group, and twenty patients into the ketamine group. At the conclusion of the study, one patient from the control group and three patients from the ketamine group were excluded from the study leaving seventeen patients in the ketamine group and eighteen patients in the standard of care group (Figure 1). In regard to the excluded patient in the control group, after randomization the patient had insurance issues, and the surgery was cancelled. In the ketamine group, one patient was discovered to have exclusion criteria on the day of surgery (substance abuse) that was not disclosed prior to randomization. The second excluded ketamine patient was unable to participate in the study following randomization due to a temporary suspension of funding (for financial reasons) from our institution that occurred during his surgical date. The third excluded ketamine patient was discovered to have exclusion criteria in the preop area (elevated intraocular pressure) following randomization. Perioperative and postoperative data was not collected for the excluded patients.

### 3.1. Demographic Data

Demographic information is shown in Table 1. Age, American Society of Anesthesiologists (ASA) physical status, gender distribution, and BMI were similar between the ketamine and control groups. Medical comorbidities are listed in Table 2. The ketamine group had a higher presence of osteoarthritis (35.3% versus 5.6%, *P*=0.03). Diabetes was present more in the ketamine group, but it was not statistically significant (47.1% versus 16.7%, *P*=0.053). For other medical conditions including the presence of chronic pain, there were no statistically significant differences between the ketamine and control groups, and no patients in either group were on chronic opioid therapy. Average ketamine infusion rate was 12.3 mg/hr, and the mean total dose received was 248.8 mg. Average duration of infusion was 23.2 hours. Ketamine infusion was not terminated early in any study patient. The bypass procedure was converted to open in two patients in the control group and three patients in the ketamine group, but this difference was not statistically significant (*P*=0.63).

### 3.2. Morphine Equivalent Consumption, Pain Scores, and Patient Satisfaction

Table 3 shows opioid consumption reported in oral morphine equivalents. Total perioperative opioid consumption was significantly lower in the ketamine group versus the control group (179.9 ME versus 248.7 ME, *P*=0.03). PCA opioid administration was also significantly lower in the ketamine group (90.6 ME versus 137.6 ME, *P*=0.04). There was a nonstatistically significant trend towards lower intraoperative opioid administration in the ketamine group (*P*=0.1).  Table 4 shows average reported pain scores at 0–12 hours, 12–24 hours, and 24–28 hours postoperatively. Pain scores were overall lower in the ketamine group, but the differences were not statistically significant.

### 3.3. Adverse Events and Side Effects

There were no adverse respiratory events, prolonged sedation, delirium, diplopia, naloxone use, pruritus, hallucinations, or adverse hemodynamic events recorded in either group.  Table 5 lists the adverse event and side effects data. In regard to postoperative nausea and vomiting (PONV), there were no statistically significant differences between the groups. There was a nonsignificant increase in length of stay for the ketamine group (43.9 hours versus 36.6 hours, *P*=0.18). There were two readmissions from the control group and none from the ketamine group; this difference was not statistically significant and deemed to be related to factors other than analgesia.

### 3.4. Patient Satisfaction Questionnaires

**Figure 2:**
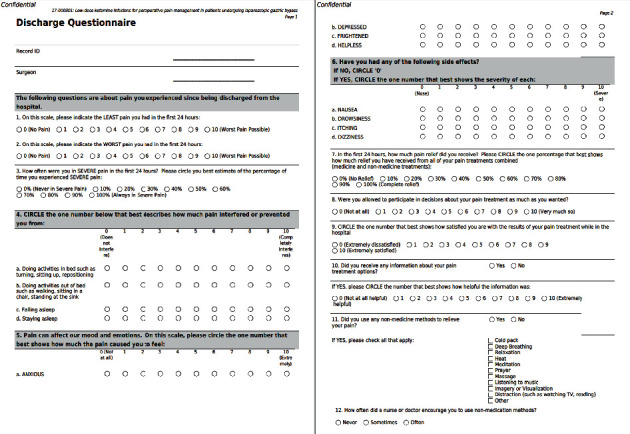
The postop questionnaire/survey given to patients at their first postop follow-up appointment since being discharged from the hospital.

A discharge questionnaire (Figure 2) was given to study participants at the time of hospital discharge. Maximum pain scores in the first 24 hours were lower in the ketamine group (5.9 versus 7.8, *P*=0.0475). There was a statistically significant difference in total percentage of pain relief in the first 24 hours within ketamine group indicating a greater percentage of overall relief (80% versus 70%, *P*=0.027). The ketamine group also had a statistically significant increase in overall satisfaction with treatment of their pain while in the hospital (9.5 versus 8.3, *P*=0.03). There was a statistically significant increase in how helpful the ketamine group patients found the information they were given regarding their pain treatment options while in the hospital when compared to the standard therapy group (9.6 versus 7.4, *P*=0.0013). There was no reported difference between how the groups used alternative methods (deep breathing, listening to music, prayer, etc.) to relieve their pain. There was no statistically significant difference in reported effect of pain on subjective measures such as sleep, mood, anxiety, nausea, drowsiness, itching, or dizziness between the two groups.

**Figure 3:**
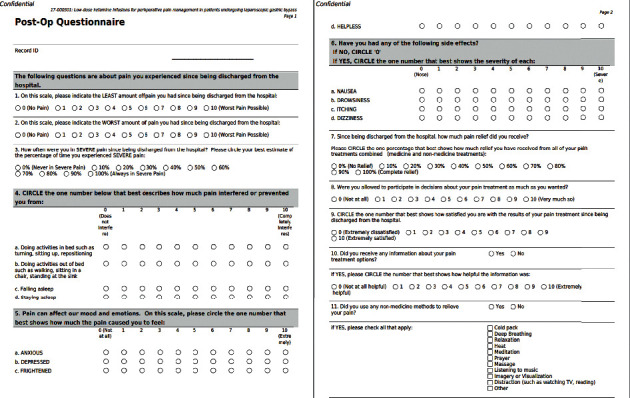
The postop questionnaire/survey given to patients at their first postop follow-up appointment since being discharged from the hospital.

An additional questionnaire (Figure 3) was given to patients at the time of their first postoperative visit with the surgeon. Maximum pain scores since being discharged from the hospital were significantly lower in the ketamine group (3.6 versus 6.2, *P*=0.0127). Regarding how much pain interfered or prevented patients from doing activities in bed such as turning, sitting up, or repositioning since being discharged, the ketamine group indicated a statistically significant lower score than the standard care group on a 10-point scale (1.0 versus 3.7, *P*=0.0005). Similarly, the ketamine group reported a statistically significant lower score in regard to how much pain interfered or prevented them from doing activities out of bed such as walking, sitting in a chair, or standing (0.9 versus 3.4, *P*=0.0009). Lastly, there was a statistically significant increase in how helpful the ketamine group patients found the information they were given regarding their pain treatment after discharge when compared to the standard therapy group (10 versus 7.9, *P*=0.0014).

## 4. Protocol Violations

### 4.1. Protocol Violations Are Listed below according to Each Group

#### 4.1.1. Control Group

One patient in the control therapy group was given a ketamine bolus after induction and received a morphine PCA instead of hydromorphone. The oral morphine equivalence data was calculated for this patient and included in the study. Preoperatively, one patient received celecoxib 400 mg and gabapentin 600 mg in addition to the standard care. Intraoperatively, two patients were given vecuronium for maintenance of paralysis. Postoperatively, three patients in this group did not receive postop ketorolac, two patients did not receive postop acetaminophen, one patient received the acetaminophen as per os (PO) instead of IV, and one patient got a dose of methocarbamol IV in the PACU. We did not exclude the patients from the data analysis as we did not think these variances compromised the integrity of the data.

#### 4.1.2. Ketamine Group

One patient in the ketamine group had an inaccurately programmed ketamine infusion pump rate by pharmacy, leading to the patient getting 1.4 mg more ketamine than was originally planned over the course of 24 hours (which represents a 0.006% increase from the dose they should have received). This same patient was also given 0.8 mg/kg bolus of ketamine (IBW) instead of 0.3 mg/kg IBW on induction. This was reported to the IRB and deemed noncompliance. In regard to deviations from postop care, three patients did not complete the scheduled regimen of ketorolac and acetaminophen as ordered overnight. One of these patients was also given one dose of methocarbamol IV in PACU. Additionally, one patient in the ketamine group was given a fentanyl PCA instead of hydromorphone. The oral morphine equivalence data was calculated for this patient and included in the study. In regard to preop medications, one patient was given 600 of gabapentin in preop. Lastly, in regard to intraop care, two patients were given vecuronium for maintenance of paralysis, and one patient was maintained on isoflurane instead of sevoflurane. Again, we did not exclude the patients from the data analysis as we did not think that these variances compromised the integrity of the data.

## 5. Discussion

In this study we demonstrate that a low-dose ketamine bolus (0.3 mg/kg) plus postoperative infusion (0.2 mg/kg/hr) reduces postoperative opioid consumption in obese patients undergoing laparoscopic gastric bypass. Patients receiving ketamine infusion averaged 179.9 ME versus 248.7 in the control group, a difference that is quantitatively substantial and statistically significant (*P*=0.03). There was a nonsignificant trend toward lower pain scores in the ketamine group suggesting that, at the very least, the lower opioid consumption did not result in worse analgesia for patients receiving ketamine. Looking at the satisfaction questionnaires, the ketamine group had lower reported worst pain scores in the first 24 hours (5.9 versus 7.8, *P*=0.0475), lower maximum pain scores while recovering after discharge (3.6 versus 7.8, *P*=0.0127), and a greater overall reported total percentage of pain relief in the first 24 hours (80% versus 70%, *P*=0.027).

Although the clinical significance of these differences is not clear, one could speculate that these lower pain scores lead to various positive outcomes seen on the discharge and follow-up questionnaires. Higher patient satisfaction with hospital pain treatment (9.5 versus 8.3, *P*=0.03) and improved ability to perform activities of daily living (ADL) postdischarge both in and out of bed were both statistically significant outcomes seen within the ketamine group. Additionally, there were no differences in incidence of adverse events, side effects, or excessive sedation for patients receiving ketamine compared with the control group both in the hospital and after discharge.

Previous investigations of ketamine for patients undergoing bariatric surgery have yielded mixed results. In a randomized comparative trial, Sollazzi et al. demonstrated that combination of S (+) ketamine 0.5 mg/kg and clonidine 3 ug/kg administered prior to open bariatric surgery reduced pain scores for 6 hours after surgery as well as intraoperative fentanyl requirements and administration of tramadol in the recovery area [11]. At 12 hours, there was no difference in pain scores, and it is impossible to determine how much of the analgesic benefits were due to ketamine as opposed to clonidine, an alpha-2 agonist which also has analgesic properties. Kasputye et al. conducted a randomized, double-blinded trial comparing preincision ketamine 0.15 mg/kg with placebo in patients undergoing bariatric surgery and demonstrated a mild but statistically significant reduction in postoperative morphine administration but no difference in pain scores [12]. In a randomized double-blinded study of patients undergoing laparoscopic gastric bypass, Wang et al. failed to find any significant difference in postoperative pain scores following a one-time bolus of ketamine 0.4 mg/kg in the recovery room [13]. The authors did find a statistically significant improvement in mood as measured by a validated pain questionnaire, an interesting finding given ketamine's known antidepressant effects [13].

Based on a previous study of 384 patients undergoing laparoscopic gastric bypass by Horsely et al., we would expect a perioperative ME of around 195–256 with a mean of 225 [14]. This amount is supported by our study. In regard to expected opioid reduction, consensus guidelines from the ASA and American Society of Pain Medicine suggest that you can see up to a 20% reduction in ME in patients receiving ketamine, another value that is supported by our study [15].

The data in the present study demonstrated a clear benefit in terms of reduced opioid consumption. The logical next question is the following: does reduced opioid consumption translate into clinically meaningful benefits for obese patients undergoing bariatric surgery? Clearly, adverse respiratory events are the most feared risk of opioid therapy for obese patients after anesthesia and surgery. In this study, there were no serious adverse events in either group. That finding is both unsatisfying and reassuring. Given the known safety profile of ketamine vis-à-vis the respiratory/pulmonary system for a heterogeneous surgical population, the current study provides additional support to ketamine's general safety profile. In addition, when combined with the data reported in the previously noted studies on ketamine and bariatric surgery, it further supports the proposition that ketamine infusions are safe for that specific population. Nonetheless, it would be compelling if it could be shown that ketamine actually reduced adverse respiratory events when compared with opioids. Unfortunately, it may be that ketamine exerts no beneficial effect or that the current study is simply underpowered to detect a difference in these fortunately rare events.

Our study did not demonstrate any benefit of ketamine in terms of reducing opioid-related sedation, nausea, or pruritis. Ileus and time to ambulation were not studied directly, though length of stay was somewhat longer in the ketamine group, suggesting that discharge-delaying ileus or inability to ambulate were not common features of the more opioid-heavy control group. Nonetheless, studies of ketamine in other populations have demonstrated reduction in opioid-related side effects such as nausea and pruritus, so it is logical to assume that these benefits would extend to bariatric patients. Ileus is a very well-known effect of opioids and is one of the primary outcomes driving the push for opioid reduction in enhanced recovery protocols for gastrointestinal surgery [16]. Again, the study was powered to evaluate opioid consumption, and it is possible that a larger study would have detected a difference in the incidence of ileus. Sedation is a more complicated question because though most prospective trials demonstrate no risk of sedation with perioperative ketamine, two retrospective studies suggest that postoperative ketamine infusions may pose a risk of sedation [17–19]. Nevertheless, in this study, ketamine infusions did not increase or decrease the incidence of sedation. It is still unclear whether replacing some amount of postoperative opioids with ketamine reduces the risk of sedation in surgical patients.

Development of postoperative opioid dependence was not evaluated in this study though it is increasingly recognized as a serious problem and an important argument for limiting opioid administration to surgical patients where possible. Bariatric surgery patients are at significant risk of developing chronic postsurgical pain and increased and prolonged postoperative opioid use is a problem in this population [20, 21]. Though ketamine has not clearly demonstrated efficacy for preventing postoperative opioid dependence, mechanistically it does block opioid-induced hyperalgesia as well as acute opioid tolerance. In addition, meta-analysis shows that ketamine may reduce the development of persistent postsurgical pain which is a risk factor for opioid overuse and dependence. Furthermore, though not proven, it is possible that by simply reducing opioid exposure, ketamine may reduce the incidence of postoperative opioid use and development of dependence. Thus, perioperative ketamine could theoretically reduce the incidence of chronic pain and opioid overuse in patients undergoing bariatric surgery.

Though perioperative ketamine does have known side effects, most commonly hallucinations, dysphoria, dizziness, and diplopia, these side effects were not seen in this study [8, 18]. The lack of side effect in this study may be due to the relatively small number of patients, modest average ketamine dose (12 mg/hr on average), and the low incidence of chronic pain, substance abuse, and psychiatric comorbidity. Despite absence in this study, bariatric patients are almost certainly still at risk for ketamine-related side effects.

The strengths of our study include the prospective study design, randomization, and standardization of our preop, intraop, and postop protocols in addition to well-matched patient cohorts. There was a clear demonstration of reduced opioid use perioperatively in the ketamine group which was our primary outcome. Interestingly, the questionnaire data suggested increased patient satisfaction with their pain control and also reported lower maximum pain scores in the ketamine group both postoperatively and after discharge. There also seemed to be a benefit in the questionnaire data in regard to decreased limitation of ADLs within the ketamine group once discharged home, although the clinical significance of the difference is unclear. More studies are needed to reliably and accurately elucidate that type of postoperative data.

Limitations of this study included being an unblinded study, which may have skewed providers willingness to give opioids, and we see a trend towards that in the intraop data. Additionally, as the PCA is patient controlled, placebo effect cannot be ruled out as the cause for the decreased opioid consumption seen in the ketamine group. Our study also showed that gastric bypass does not guarantee a diagnosis of OSA. Therefore, this study should not be used to prove that ketamine is safe in patients with OSA. Additionally, our study was small, which inevitably makes it difficult to detect rare adverse events, and it was only powered to detect a difference in perioperative opioid use. There was a very low prevalence of chronic pain/opioid tolerant patients in this study, making it difficult to draw any conclusions about decreased opioid use for this type of patient population undergoing gastric bypass surgery. Furthermore, there were a number of protocol violations, likely secondary to the variety of providers involved in the study. Standardizing the providers for every case was simply not reasonable given the scheduling variation. However, despite these violations, we do not feel that there was a significant difference between the types/severity of violations in each group which could potentially explain any significant differences in results between the two groups. Lastly, there were four patients who were randomized but then excluded in the analysis as mentioned previously. In these cases, after discussion with our statisticians, an intention-to-treat analysis was not done because two of the four patients met exclusion criteria after randomization, and no data was available for the other two patients.

## 6. Conclusion

Overall, our study showed a statistically significant decrease in perioperative opioid use in obese patients undergoing laparoscopic gastric bypass surgery when given a 0.3 mg/kg bolus (IBW) followed by a low-dose infusion of 0.2 mg/kg/hour (IBW) which continued into the postoperative period. There was no statistically significant difference in pain scores or length of stay postoperatively between the two groups and no reported adverse respiratory events, prolonged sedation, agitation, or other side effects. Although the patient satisfaction questionnaires showed a significant difference in the ketamine group in regard to lower reported maximum pain scores, a reported relative improvement in how pain limited ADLs once discharged, and an increased overall satisfaction with how pain was managed in the hospital, the clinical significance of these observed differences is unclear, and further studies are needed to clarify these findings.

## Figures and Tables

**Table 1:** Lists demographic data between the groups.

Demographic information between groups				
	C	K	Total	*P* value
	(*N* = 18)	(*N* = 17)	(*N* = 35)	
Age				0.1877^2^
N	18	17	35	
Mean (SD)	49.3 (10.1)	54.2 (11.5)	51.7 (10.9)	
Range	(30.0–65.0)	(33.0–69.0)	(30.0–69.0)	

ASA				0.0928^2^
N	18	17	35	
Mean (SD)	2.6 (0.5)	2.8 (0.4)	2.7 (0.5)	
Range	(2.0–3.0)	(2.0–3.0)	(2.0–3.0)	

BMI				0.5152^2^
N	18	17	35	
Mean (SD)	45.8 (5.1)	44.7 (5.0)	45.3 (5.0)	
Range	(39.4–58.4)	(37.3–53.3)	(37.3–58.4)	

Gender				0.1761^1^
F	16 (88.9%)	12 (70.6%)	28 (80.0%)	
M	2 (11.1%)	5 (29.4%)	7 (20.0%)	

^1^Chi-square. ^2^Equal variance *t*-test. ASA = American Society of Anesthesiologists physical classification status and BMI = body mass index.

**Table 2:** Medical comorbidity data between the two therapy groups.

Medical comorbidities between groups				
	C	K	Total	*P* value
	(*N* = 18)	(*N* = 17)	(*N* = 35)	
A-fib				0.2965^1^
N	18 (100.0%)	16 (94.1%)	34 (97.1%)	
Y	0 (0.0%)	1 (5.9%)	1 (2.9%)	

Asthma				0.5808^1^
N	16 (88.9%)	16 (94.1%)	32 (91.4%)	
Y	2 (11.1%)	1 (5.9%)	3 (8.6%)	

CAD				0.3241^1^
N	17 (94.4%)	17 (100.0%)	34 (97.1%)	
Y	1 (5.6%)	0 (0.0%)	1 (2.9%)	

Chronic pain				0.5119^1^
N	17 (94.4%)	15 (88.2%)	32 (91.4%)	
Y	1 (5.6%)	2 (11.8%)	3 (8.6%)	

COPD				0.2965^1^
N	18 (100.0%)	16 (94.1%)	34 (97.1%)	
Y	0 (0.0%)	1 (5.9%)	1 (2.9%)	

CPAP				0.5808^1^
N	8 (44.4%)	6 (35.3%)	14 (40.0%)	
Y	10 (55.6%)	11 (64.7%)	21 (60.0%)	

Depression				0.7738^1^
N	13 (72.2%)	13 (76.5%)	26 (74.3%)	
Y	5 (27.8%)	4 (23.5%)	9 (25.7%)	

GERD				0.6005^1^
N	9 (50.0%)	10 (58.8%)	19 (54.3%)	
Y	9 (50.0%)	7 (41.2%)	16 (45.7%)	

HF				0.2965^1^
N	18 (100.0%)	16 (94.1%)	34 (97.1%)	
Y	0 (0.0%)	1 (5.9%)	1 (2.9%)	

HLD				0.3576^1^
N	10 (55.6%)	12 (70.6%)	22 (62.9%)	
Y	8 (44.4%)	5 (29.4%)	13 (37.1%)	

HTN				0.2291^1^
N	10 (55.6%)	6 (35.3%)	16 (45.7%)	
Y	8 (44.4%)	11 (64.7%)	19 (54.3%)	

Morbid obesity				
Y	18 (100.0%)	17 (100.0%)	35 (100.0%)	

OA				0.0279^1^
N	17 (94.4%)	11 (64.7%)	28 (80.0%)	
Y	1 (5.6%)	6 (35.3%)	7 (20.0%)	

OSA				0.8259^1^
N	7 (38.9%)	6 (35.3%)	13 (37.1%)	
Y	11 (61.1%)	11 (64.7%)	22 (62.9%)	

PONV				0.9668^1^
N	17 (94.4%)	16 (94.1%)	33 (94.3%)	
Y	1 (5.6%)	1 (5.9%)	2 (5.7%)	

T2DM				0.0529^1^
N	15 (83.3%)	9 (52.9%)	24 (68.6%)	
Y	3 (16.7%)	8 (47.1%)	11 (31.4%)	

^1^Chi-square test. A-fib = atrial fibrillation, CAD = coronary artery disease, COPD = chronic obstructive pulmonary disease, CPAP = continuous positive airway pressure device use, GERD = gastroesophageal reflux disease, HF = heart failure, HLD = hyperlipidemia, HTN = hypertension, OA = osteoarthritis, OSA = obstructive sleep apnea, PONV = postoperative nausea and vomiting, and T2DM = type 2 diabetes mellitus.

**Table 3:** Data on opioid consumption in oral morphine equivalents.

Opioid analysis ketamine versus standard therapy				
	Ketamine	Standard therapy	Total	*P* value
	(*N* = 17)	(*N* = 18)	(*N* = 35)	
PCA				0.0458^1^
N	17	18	35	
Mean (SD)	90.6 (76.5)	137.6 (75.2)	114.8 (78.4)	
Range	(0.0–248.0)	(40.8–315.6)	(0.0–315.6)	

OR				0.1095^1^
N	17	18	35	
Mean (SD)	57.8 (22.8)	72.5 (23.9)	65.4 (24.2)	
Range	(30.0–90.0)	(30.0–135.0)	(30.0–135.0)	

Post				0.3368^1^
N	17	18	35	
Mean (SD)	31.4 (25.2)	38.6 (22.2)	35.1 (23.6)	
Range	(0.0–89.0)	(7.5–75.0)	(0.0–89.0)	

Total				0.0282^1^
N	17	18	35	
Mean (SD)	179.9 (113.1)	248.7 (88.8)	215.3 (105.7)	
Range	(51.1–427.0)	(139.5–450.6)	(51.1–450.6)	

^1^Equal variance *t*-test. PCA: patient-controlled analgesia pump; OR: operating room; and Post: PO pain meds given in the postoperative period.

**Table 4:** Pain score data between the groups.

Pain scores between groups				
	C	K	Total	*P* value
	(*N* = 18)	(*N* = 17)	(*N* = 34)	
Avg. pain score 0–12 hours				0.1613^1^
N	18	17	35	
Mean (SD)	4.4 (1.5)	3.4 (2.2)	3.9 (1.9)	
Range	(2.0–7.0)	(0.0–7.4)	(0.0–7.4)	
Difference (95% CI)			1.0 (–0.3, 2.3)	

Avg. pain score 12–24 hours				0.0585^1^
N	18	17	35	
Mean (SD)	3.7 (1.3)	2.6 (1.9)	3.1 (1.7)	
Range	(1.4–7.2)	(0.0–6.9)	(0.0–7.2)	
Difference (95% CI)			1.1 (0, 2.2)	

Avg. pain score 24–48 hours^∗^				0.2559^1^
N	17	16	33	
Mean (SD)	3.8 (1.4)	3.1 (1.9)	3.4 (1.7)	
Range	(1.0–6.4)	(0.0–6.6)	(0.0–6.6)	
Difference (95% CI)			0.7 (−0.4, 1.8)	

^1^Chi-square. ^2^Equal variance *t*-test. Avg.: average and CI: confidence interval. ^∗^1 patient from each group was discharged early so this value was not obtained in 2 patients.

**Table 5:** Data on the adverse events and side effects that occurred.

Adverse events and side effects				
	C	K	Total	*P* value
	(*N* = 18)	(*N* = 17)	(*N* = 35)	
Avg. RASS 1st 12 hours				0.1973^1^
Number	18	17	35	
Mean (SD)	−0.6 (0.3)	−0.5 (0.4)	−0.6 (0.4)	
Range	(−1.1–0.0)	(−1.8–0.3)	(−1.8–0.3)	

Avg. RASS 2nd 12 hours				0.7869^1^
Number	18	17	35	
Mean (SD)	0.0 (0.1)	0.0 (0.2)	0.0 (0.1)	
Range	(−0.5–0.0)	(−0.7–0.0)	(−0.7–0.0)	

Avg. RASS 2nd 24 hours				1.0000^1^
N	18 (100%)	17 (100%)	35 (100%)	

Agitation 1st 12 hours				1.0000^1^
N	18 (100%)	17 (100%)	35 (100%)	

Agitation 2nd 12 hours				1.0000^1^
N	18 (100%)	17 (100%)	35 (100%)	

Agitation 2nd 12 hours				1.0000^1^
N	18 (100%)	17 (100%)	35 (100%)	

Adverse hemodynamic events				1.0000^1^
N	18 (100%)	17 (100%)	35 (100%)	

Delirium 1st 12 hours				1.0000^1^
N	18 (100%)	17 (100%)	35 (100%)	

Delirium 2nd 12 hours				1.0000^1^
N	18 (100%)	17 (100%)	35 (100%)	

Delirium 2nd 12 hours				1.0000^1^
N	18 (100%)	17 (100%)	35 (100%)	

Diplopia 1st 12 hours				1.0000^1^
N	18 (100%)	17 (100%)	35 (100%)	

Diplopia 2nd 12 hours				1.0000^1^
N	18 (100%)	17 (100%)	35 (100%)	

Diplopia 2nd 12 hours				1.0000^1^
N	18 (100%)	17 (100%)	35 (100%)	

Hallucinations 1st 12 hours				1.0000^1^
N	18 (100%)	17 (100%)	35 (100%)	

Hallucinations 2nd 12 hours				1.0000^1^
N	18 (100%)	17 (100%)	35 (100%)	

Hallucinations 2nd 12 hours				1.0000^1^
N	18 (100%)	17 (100%)	35 (100%)	

Respiratory depression 1st 12 hours				1.0000^1^
N	18 (100%)	17 (100%)	35 (100%)	

Respiratory depression 2nd 12 hours				1.0000^1^
N	18 (100%)	17 (100%)	35 (100%)	

Respiratory depression 2nd 12 hours				1.0000^1^
N	18 (100%)	17 (100%)	35 (100%)	

Nausea 1st 12 hours				0.8452^1^
N	8 (44.4%)	7 (41.2%)	15 (42.9%)	
Y	10 (55.6%)	10 (58.8%)	20 (57.1%)	

Nausea 2nd 12 hours				0.2076^1^
N	15 (83.3%)	11 (64.7%)	26 (74.3%)	
Y	3 (16.7%)	6 (35.3%)	9 (25.7%)	

Nausea 2nd 24 hours				0.9387^1^
N	15 (83.3%)	14 (82.4%)	29 (82.9%)	
Y	3 (16.7%)	3 (17.6%)	6 (17.1%)	

Length of stay (hours)				0.1824^2^
Number	18	17	35	
Mean (SD)	36.6 (13.6)	43.9 (18.0)	40.1 (16.1)	
Range	(24–72.5)	(28.0–102.0)	(24.0–102.0)	

Pruritus				1.0000^1^
N	18 (100%)	17 (100%)	35 (100%)	

Readmission				0.1570^1^
N	16 (88.9%)	17 (100.0%)	33 (94.3%)	
Y	2 (11.1%)	0 (0.0%)	2 (5.7%)	

Lists data on the adverse events and side effects that occurred. Avg: average; Resp: respiratory; and RASS: Richmond agitation-sedation scale.

## Data Availability

The datasets generated and analyzed during the current study are available from the corresponding author upon reasonable request.

## Abbreviations

ADL: Activities of daily living
ASA: American Society of Anesthesiologists
BMI: Body mass index
FRC: Functional residual capacity
IBW: Ideal body weight
IV: Intravenous
ME: Morphine equivalents
NMDA: N-Methyl-D-aspartate
OSA: Obstructive sleep apnea
PACU: Postanesthesia care unit
PCA: Patient-controlled analgesia
PO: Per Os
PONV: Postoperative nausea and vomiting
RASS: Richmond agitation-sedation scale.
